# Prevalence and Levels of Symptoms of Anxiety, Depression and Suicidal Ideation Amongst Elite Male Soccer Players: An Age- and Education-Matched Controlled Study

**DOI:** 10.3390/ijerph23030362

**Published:** 2026-03-12

**Authors:** Gisele Maria Rosa Sobrinho, Heloísa Gonçalves Ferreira, David R. McDuff, Alberto Filgueiras

**Affiliations:** 1 Departamento de Desenvolvimento e Cognição, Instituto de Psicologia, Universidade do Estado do Rio de Janeiro, Rio de Janeiro 20550-900, RJ, Brazil; 2 Department of Psychiatry, University of Maryland School of Medicine, Baltimore, MD 21201, USA; 3 College of Psychology, School of Health, Medical and Applied Sciences, Central Queensland University, Cairns 4780, Australia; 4 S.P.O.R.T. Research Cluster, Central Queensland University, Rockhampton, QLD 4701, Australia

**Keywords:** mental health, prevalence, soccer, anxiety, depression

## Abstract

**Highlights:**

**Public health relevance—How does this work relate to a public health issue?**

- Mental health symptoms, including anxiety and depression, are prevalent among elite male soccer players at similar levels compared to controls.
- The use of matched control groups with standardised assessment measures addresses a critical gap in population-based mental health research involving elite footballers (soccer players).

**Public health significance—Why is this work of significance to public health?**

- Findings from this study challenge assumptions that elite sports are inherently protective against mental health difficulties, particularly suicidal ideation.
- Although anxiety and depression levels were comparable between groups, higher suicidal ideation among elite soccer players highlights ongoing public health concerns for athlete mental health.

**Public health implications—What are the key implications or messages for practitioners, policy makers and/or researchers in public health?**

- Mental health monitoring in elite sports should prioritise ongoing, validated multidimensional assessment tools rather than reliance on diagnostic thresholds alone.
- Public health strategies targeting athletes’ mental health support should explicitly include suicide prevention and tailored psychological interventions within high-performance football clubs and organisations.

**Abstract:**

Mental health among elite athletes has received increasing attention, yet evidence from studies including matched control groups remains limited. This study investigated the prevalence and levels of anxiety, depression, and suicidal ideation among elite male soccer players compared with age- and education-matched controls from the general population. A total of 324 participants were included, comprising 214 elite male soccer players and 110 controls. Participants completed the Beck Depression Inventory–II (BDI-II) and the Beck Anxiety Inventory (BAI). Suicidal ideation was assessed using item 9 of the BDI-II. Data were analysed using descriptive statistics, reliability analysis, and non-parametric inferential statistics. In addition, a neural network classification analysis was conducted to examine whether combined anxiety and depressive symptoms could differentiate elite athletes from controls. No significant differences were found in the prevalence of depression (40.19% in athletes vs. 37.27% in controls) or anxiety (87.38% in athletes vs. 90.00% in controls). Levels of depressive symptoms were similar between groups, whereas anxiety levels were higher among controls. Suicidal ideation was significantly less prevalent in controls (22.73%) compared with elite soccer players (46.73%). Neural network classification achieved above-chance accuracy (68.8%) in differentiating athletes based on combined anxiety and depressive symptoms, but low sensitivity reinforces prior results that athletes and matched controls have similar levels of mental health outcomes. Elite soccer players and controls show similar prevalence of depression and anxiety, although anxiety severity appears lower among athletes. The joint configuration of anxiety and depressive symptoms modestly differentiates group affiliation, while suicidal ideation remains more prevalent among elite players. These findings highlight the complex and distinctive mental health profile of elite athletes and suggest the need for continued psychological support and monitoring in high-performance sport contexts.

## 1. Introduction

Mental health amongst elite athletes has been a topic of debate in sport psychology and psychiatry in the past decades, with increased supporting evidence to best draft and provide services to these individuals. Research indicates that elite athletes are not immune to mental health symptoms, with studies showing that rates of anxiety and depression among athletes are comparable to those in the general population [1,2]. A systematic review highlighted that elite athletes experience significant levels of mental illness symptoms, which can be fuelled by the pressures of competitions and the demands of high-performance sports [3]. Extensive training loads associated with competitive sport present potential threats to athletes’ mental health, leading to increased vulnerability to mental health disorders [4].

The relationship between mental health and sport performance in elite athletes is highly complex. Mental health symptoms and disorders potentially impair athletic performance and increase the risk of physical injuries [2,5]. The International Olympic Committee (IOC) has recognised the importance of addressing mental health symptoms in athletes, proposing a comprehensive mental health plan for constant assessments and interventions [2,6]. A review revealed that the prevalence of anxiety and depression among elite athletes ranges from 16% to 34%, with former athletes also exhibiting high rates of distress [7]. In contrast, control groups, which typically include non-athletes, show varied prevalence rates, often lower than those observed in elite athletes [8]. For instance, while elite athletes may report anxiety symptoms at rates as high as 34%, the general population’s prevalence is generally lower, indicating a heightened vulnerability among athletes [7,9].

One dangerous mental health symptom that needs to be addressed is suicidal ideation. Prevalence studies of suicidal ideation amongst elite athletes suggest rates that can be alarmingly high [10]. For instance, a study by Sun et al. [10] reported that approximately 18% of elite athletes experienced suicidal ideation. This finding is consistent with the work of Timpka et al. [11], who found that about 15.6% of track and field athletes reported having experienced suicidal thoughts, and Ojio et al. [12], who reported that 7.6% of rugby elite players presented suicidal-related symptoms within a two-week period of longitudinal data collection. A meta-analysis conducted by Gill et al. [13] suggested that athletes show lower suicidal thoughts and attempts than non-athletes; however, half of the included studies compared University athletes with their non-athletic peers, and only one study included elite athletes showing non-significant differences between them and controls [14]. Lindqvist et al. [15] compared suicide deaths amongst International or Olympic power sports (i.e., wrestling, powerlifting and throwing events in track and field) and showed higher rates amongst athletes when compared to the general population of men.

Anxiety and depressive symptoms are among the most frequently reported mental health outcomes in research on elite athletes. Prevalence estimates vary widely across sports, competitive levels, and methodological approaches. Recent reviews indicate that elite athletes experience rates of anxiety and depression that are comparable to, and in some cases higher than, those observed in the general population, particularly during periods of injury, deselection, performance failure, or career transition [1,2,6]. The elite sport context is characterised by performance pressure, public evaluation, job instability, and limited recovery opportunities, all of which may contribute to sustained psychological distress [3,4]. Accordingly, anxiety and depression are not only relevant outcomes on their own, but constitute well-established risk factors for suicidal ideation, especially when symptoms are persistent and embedded within high-demand environments. By looking at anxiety and depressive symptoms alongside suicidal ideation, this study aims to provide an essential understanding of mental health risk profiles in elite soccer players while directly informing this study’s hypotheses and analytical strategies.

Studies on the general population suggest that the prevalence of suicidal ideation is generally similar to that of athletes [16,17,18,19]. A systematic review indicated that the pooled prevalence of suicidal ideation in the general population during the early stages of the COVID-19 pandemic was approximately 11.5% [16]. This increase in suicidal thoughts during the pandemic aligns with findings from other studies, which reported a lifetime prevalence of suicidal ideation at 9.2% across 17 countries [18]. In Belgium, a national health survey revealed that 3.3% of the population seriously contemplated suicide in the past year, highlighting the variability in rates based on geographic and temporal contexts [19]. Additionally, a representative survey in Germany found a prevalence of 8.0% for suicidal ideation within the last two weeks, suggesting that short-term assessments may yield higher rates than lifetime prevalence estimates [17].

In summary, there is no consensus whether levels of anxiety or depression symptoms amongst elite athletes are higher, lower or the same in comparison to control groups or the general population [1,2,7,8,9]. Based on prevalence studies across the globe, evidence suggests that suicidal ideation amongst elite athletes, from 7.6% to 18% [10,11,12], might be either higher or similar when compared to the general population, from 3.3% to 11.5% [16,17,18,19]. While suicidal thoughts do not differ statistically between elite athletes and control groups [14], suicide deaths seem higher amongst international-level athletes [15].

Even though there is evidence on the prevalence and levels of mental health symptoms amongst elite athletes, only a few studies have utilised control groups. Control groups are important in this type of research because they provide a baseline for comparison, help control for confounding variables and enhance the validity of the findings [20]. The absence of control groups in research with elite athletes is a key point highlighted by Gouttebarge et al. [7] in their review and meta-analysis. Thus, there is a gap in the literature when it comes to mental health symptoms with supporting evidence from controlled studies. This gap is even more pronounced in suicidal ideation, with only one study [14] that compared suicidal ideation between international-level athletes and controls, finding no statistical difference; nonetheless, when University-level athletes were compared to their peers, suicidal thoughts were significantly lower amongst athletes [13].

Another gap in the literature important to highlight is mental health amongst soccer players. Soccer is one of the most celebrated sports in the world. The 2022 Qatar World Cup recorded the highest audience in the history of sports with around 5.4 billion unique viewers worldwide [21]. Gouttebarge et al. [22] found that approximately 26% of players exhibited symptoms of anxiety and depression, which was notably higher than the general population’s prevalence of around 7.6% for similar mental health issues; however, this study, such as others of the same nature, did not collect data on controls from the general population. The only study that retrieved data from both professional soccer players and their respective non-athletic counterparts in the general population was Junge and Prinz [23]. Results suggested no significant difference in either anxiety or depression symptoms; nonetheless, data were collected amongst female soccer athletes [23]. To the best of our knowledge, no study has so far studied depression and anxiety symptoms amongst male elite soccer athletes in comparison to age-matched controls from the general population.

Beyond traditional inferential group comparisons, classification approaches allow examination of whether combinations of psychological symptoms meaningfully distinguish elite athletes from matched controls at the individual level. Neural network analysis is particularly suitable for this purpose because it accommodates non-linear relationships and interactions between variables without relying on distributional assumptions. In mental health research, such approaches have been increasingly adopted to examine interacting symptom patterns that may remain obscured in univariate analyses [24,25]. Recent evidence in elite sport further supports the use of neural networks to explore group differentiation, even when psychological constructs show substantial overlap, as demonstrated by Bonnetti et al. [26] using cognitive and personality measures. In this context, neural network analysis provides a confirmatory classification strategy to assess whether anxiety and depressive symptoms jointly contribute to the differentiation between elite soccer players and matched controls.

The goal of this article is to reduce the gap of evidence in elite athletes’ mental health research by comparing levels and prevalence of depression, anxiety and suicidal thoughts amongst professional soccer players to controls with similar demographics. Based on previous findings, our initial hypothesis is that significant differences in depression and anxiety will not be found [1,2,7,9], whereas suicidal ideation will be either higher amongst controls or similar [15,16,17,18]. Levels of anxiety symptoms might be more severe amongst controls, but we do not expect differences in the severity of depressive symptoms [10,11,12,19].

## 2. Methods

### 2.1. Participants

A total of 324 individuals participated in this study. We had 214 male elite soccer players participating and 110 male volunteers from the general population within the same age and education range as the athletes. We have chosen to collect data amongst controls with matched age and education to ensure these variables did not influence the results. We recruited participants after the approval of the institutional ethics committee.

To recruit elite soccer athletes, the last author contacted sport psychologists and physical conditioning coaches of 10 clubs from the first division of the Brazilian soccer league. A total of 8 clubs responded positively. Amongst the 242 elite players recruited, 10 did not agree to participate or did not sign the terms of consent (4.13%), and 18 were excluded for not responding to the scales adequately, constituting incomplete or missing data (7.44%), who were excluded from analysis, leaving a total of 214 participants.

To recruit controls from the general population who matched the gender, age and education levels of elite soccer athletes, we advertised on social media and asked friends and colleagues to promote the study at their workplaces. We began recruiting these controls only after collecting data from the players to ensure they matched the athletes’ gender, age and education range. Inclusion criteria comprised volunteers who were adults (≥18 years old) working in full-time jobs (to mimic elite athletes’ work routine) within the age and educational ranges of the soccer players’ sample. Exclusion criteria were prior mental health or psychiatric diagnoses; physical health issues such as cardiac diseases, kidney or other chronic syndromes or disabilities and other chronic conditions; and participants, even at amateur levels, engaging in organised sports and competitions. These last criteria were screened before participants commenced responding to questionnaires, including demographics. Amongst the 156 male volunteers, 12 did not agree to participate or did not sign the terms of consent (7.69%), whereas we found incomplete or missing data from 34 of them (21.80%) who were excluded from analysis, leaving a total of 110 gender-, age- and education-matched controls. No additional demographic variables (e.g., occupation, type and frequency of exercise engagement, or detailed health history) were collected, which limits assessment of potential confounds.

Recruited participants were young adults with similar demographics across groups (athletes: age M = 25.29, SD = 3.90; education M = 7.95, SD = 1.88; controls: age M = 25.25, SD = 4.37; education M = 8.01, SD = 1.81). Most participants from both samples had completed the equivalent of high school in Brazil (i.e., 12 years of education). All soccer players were full-time professional athletes, whereas all controls were full-time workers. This decision was made to avoid external confound variables such as participants attending school, which is common amongst young adults, instead of working full-time like professional players do.

### 2.2. Procedures

This study was initially approved by the Ethics Board Committee of the Rio de Janeiro State University (Universidade do Estado do Rio de Janeiro), under substantiated report no. 2.990.087. All procedures were conducted in accordance with the ethical standards of the institutional research committee and with the Declaration of Helsinki.

After the approval of this study’s project on the ethics committee and the recruitment of participants, we sent to all volunteers the link for a web-based form prepared at Google Forms containing five pages. The first page was the study’s briefing, explaining its goals and procedures, including a brief screening for inclusion and exclusion criteria. The second page was the consent form to be virtually signed by ticking a check box to agree to participation, the participant’s email for further communication, and the demographic data collection: gender, age and education level (in school years). Education (in school years) reflects total years of formal schooling completed, as self-reported in the demographic form. Then, the third page was the Beck Depression Inventory, 2nd edition (BDI-II), followed by the Beck Anxiety Inventory (BAI) on the fourth page. The last was a thank you page including additional information on how to reach mental health practitioners if needed be, and the ethics committee and the authors’ contacts. Participants had the right to withdraw at any moment by closing the internet browser, but if they submitted, they also had the right to withdraw their data within the following 15 days after data collection.

Although the online data collection platform did not allow real-time monitoring, and automated referral prompts were not programmed, the last page of the survey provided contacts and referrals for further support, if the participant wanted to do so. In addition, all participants who endorsed any level of suicidal ideation (i.e., scores ≥ 1 on item 9 of the BDI-II) were subsequently contacted via email for further individual support. Referrals were made to a specialised mental health team composed of postgraduate health psychologists and psychiatrists affiliated with the Rio de Janeiro State University Hospital (Hospital Universitário Pedro Ernesto). Because in the final dataset all personal data and forms of identification, such as e-mail or IP address, were deleted, this procedure ensured appropriate clinical follow-up while preserving participant anonymity during initial data collection.

Data was extracted via the export tool of Google Docs to Microsoft Excel for data curation. Volunteers who did not agree to participate were automatically excluded. Participants who did not answer the demographic questions were also excluded. Participants who were screened by the inclusion and exclusion criteria were also excluded. All volunteers were male. If any item from either BDI-II or BAI was left unanswered, it was considered missing data, and the participant was excluded from the sample. Any personal information that might have led to participants’ identification, such as IP address, and time and place of the data collection, was excluded from the database. The only variables that remained for analysis were age (in years), education (in school years), condition (elite soccer players vs. controls), BDI-II and BAI responses. For the purposes of this study, the word “condition” refers to participant groups (elite soccer players vs. controls), not health conditions. Additional columns were included in the final spreadsheet due to analysis requirements, total score of BAI and BDI-II, and classification of anxiety and depression, separating minimal levels of symptoms from higher levels.

### 2.3. Instruments

The study’s briefing, consent form and demographic data (i.e., educational level and age measured in years) were presented and utilised according to the Google Forms’ format. Two standardised scales were included in the study, BDI-II and BAI.

The Beck Depression Inventory, 2nd edition (BDI-II) is a widely utilised self-report questionnaire developed to measure the severity of depressive symptoms in individuals aged 13 and older. It consists of 21 items, each corresponding to a specific symptom of depression, and respondents rate the severity of their symptoms over the past two weeks on a scale from 0 to 3, yielding a total score that ranges from 0 to 63 [27]. In this study, we used the Brazilian norms [28].

The Beck Anxiety Inventory (BAI) is a self-report questionnaire developed by Beck et al. [29], specifically designed to measure the severity of anxiety symptoms in individuals. Comprising 21 items, the BAI assesses various anxiety-related symptoms experienced over the past week, with each item rated on a scale from 0 to 3, resulting in a total score that can range from 0 to 63. In this study, the Brazilian-adapted and standardised version was used [30].

Finally, research supports the validity of using item 9 of BDI-II as an indicator of suicidal ideation. For instance, studies have shown that responses to this item correlate significantly with other standardised measures of suicidal thoughts, such as the Beck Scale for Suicide Ideation [31]. Furthermore, item 9 has been linked to various psychological symptoms, including insomnia and anxiety [32]. Because elite soccer is a time-demanding environment [23], we opted not to include further scales in this study to avoid participant drop off, adopting item 9 of BDI-II as an indicator for suicidal ideation [31,32].

### 2.4. Data Analysis

Descriptive statistics were conducted based on the nature of the data. Nominal and ordinal data were presented in terms of frequency (*N*) and percentage (%), whereas scale and continuous data were described in terms of average (M) and standard deviation (SD). Normality was tested using the Shapiro–Wilk test. The confirmation of the null hypothesis (*p* ≥ 0.05) was utilised as a reference for normality. In the case of normal distribution, Student’s *t*-test with *p*-value and Cohen’s d would be utilised to determine statistical differences and effect size. In the case of non-normal distribution, the Mann–Whitney’s U test would be adopted with *p*-value as evidence of statistical difference and the rank biserial correlation as effect size measurement.

To ensure data reliability, both BDI-II and BAI underwent a reliability analysis. We utilised the criterion of McDonnald’s omega (ω) and Cronbach’s alpha (α) higher than 0.70 as evidence of reliable data. Prevalence of anxiety, depression and suicidal ideation was determined based on the division of scores between minimal levels of symptoms and higher levels. For BDI-II, scores lower than 14 meant minimal symptoms of depression, whereas the presence of symptoms was considered when scores were equal to or above 14. For BAI, scores lower than 8 meant minimal symptoms of anxiety, whereas the presence of anxiety was determined by scores equal to or higher than 8. For suicidal ideation, we followed the procedure of Wigg et al. [32] who suggested that if participants scored 0 (zero) in this item, it meant no suicidal thoughts, whereas any other higher score would signify the presence of suicidal ideation. Prevalence was calculated by frequency and percentage of significant symptoms. To compare elite soccer players and controls, we adopted a chi-square statistic with a *p*-value lower than 0.05 as evidence of statistical significance and Cramér’s V as an indication of effect size.

**Figure 1:**
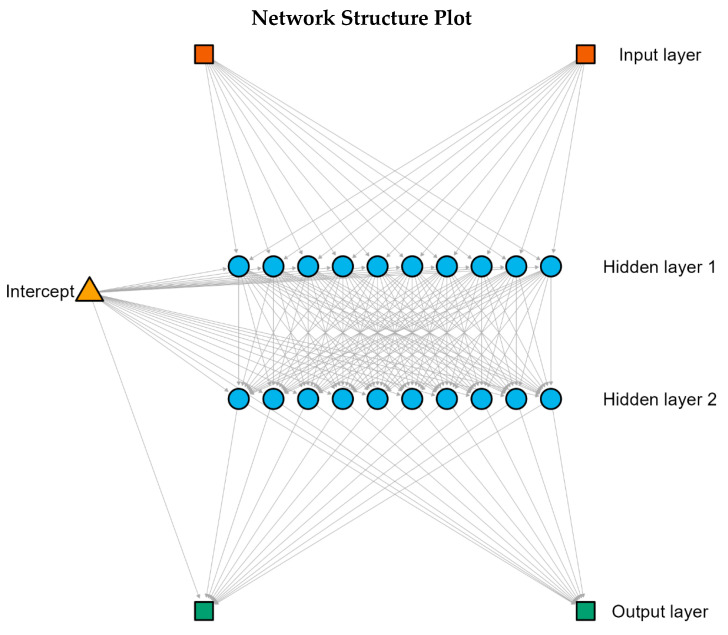
Neural network structure for classification of elite soccer players and controls. Note: Diagram of the feedforward multilayer perceptron used to classify elite soccer players and age- and education-matched controls based on combined anxiety and depressive symptom scores. Arrows represent weighted connections of forward propagation from one layer to the next through which information flows between nodes of the network. The model includes two input nodes (total BAI and BDI-II scores), two hidden layers with ten nodes each, and one output layer representing group classification. Connection weights reflect the relative contribution of each pathway to the final classification.

To confirm results from previous null-hypothesis testing, we adopted a confirmatory and supplementary analysis using a neural network classification analysis to assess whether combined anxiety and depressive symptom scores could classify participants as elite soccer players or controls. Criterion condition (elite athletes; controls) was specified as a binary categorical target variable, with total BAI and BDI-II scores entered as input. Considering the two predictors utilised in this model, two hidden layers were specified to allow hierarchical non-linear combinations of anxiety and depressive symptom scores, and ten nodes per layer were selected to provide sufficient model flexibility while retaining the same inputs, optimisation criterion, and convergence parameters. Figure 1 depicts the network structure with two hidden layers with 10 nodes each, utilised in this analysis for better convergence. The number of hidden nodes was defined to allow the model greater flexibility to capture non-linear interactions between anxiety and depressive symptoms, while maintaining the same inputs, optimisation criterion, and convergence parameters. A feedforward multilayer perceptron was estimated using logistic sigmoid activation functions and resilient backpropagation optimisation. The model was trained with a maximum of 100,000 training repetitions, with convergence determined by stabilisation of the loss function. Data were randomly split into training (80%) and test (20%) sets in accordance with Haykin [33] suggestions, with feature scaling applied prior to estimation. Neural network classification in this context was intended to assess probabilistic group separation rather than diagnostic or predictive precision, particularly given the known overlap of mental health symptom distributions between elite athletes and the general population. Model specification followed established recommendations for psychological data characterised by non-normality and potential non-linear structure [26,33,34].

Anonymized data is available at the Open Science Framework repository [35]. Data analysis was conducted in JASP 0.18.3, and syntaxes are available in the same repository under a different file: https://doi.org/10.17605/OSF.IO/TMVH9.

## 3. Results

Descriptive statistics and normality tests are presented in Table 1. Our results yielded a non-normal distribution of data for both groups, controls and athletes, in all variables, which entailed consequent non-parametric inferential statistics.

Regarding reliability, the Beck Depression Inventory, 2nd edition (BDI-II) yielded acceptable reliability with ω = 0.783 and α = 0.783; whereas the Beck Anxiety Inventory (BAI) retrieved reliability above the criterion with ω = 0.708 and α = 0.707. By ensuring reliable data, we could proceed to inferential statistics, comparing age- and education-matched controls and elite soccer athletes.

The first two comparisons were age and education. We had to make sure groups had similar results in these variables to allow comparison and avoid potential confounding variables. Neither age (U = 11,852.50; *p* = 0.918; *r* = 0.007) nor education (U = 11,605.00; *p* = 0.835; *r* = −0.014) retrieved significant differences. Thus, differences between groups in mental health outcomes cannot be explained by either of these variables.

We used the scales’ scores above the minimum cut-off points for both the BDI-II (scores higher than 13) and BAI (scores higher than 7) to assess the presence or absence of symptoms. Results for bands of severity based on cut-off points are available on Table 2. Then, we calculated percentages and conducted a chi-square test to determine whether the prevalence of symptoms was higher in one group compared to the other. For depression, prevalence amongst elite soccer players was 40.19% (86 out of 214); whereas the control group showed a prevalence of 37.27% (41 out of 110). The chi-square comparison between elite soccer athletes and controls yielded non-significant differences with a negligible effect size (χ^2^ = 0.259; *p* = 0.611; Cramér’s V = 0.028). For anxiety, prevalence amongst elite soccer athletes was 87.38% (187 out of 214); whereas prevalence of anxiety symptoms amongst controls was 90.00% (99 out of 110). The chi-square revealed a lack of statistical significance with a negligible effect size (χ^2^ = 0.481; *p* = 0.488; Cramér’s V = 0.039).

Prevalence of suicidal ideation was calculated based on BDI-II item 9. The presence or absence of suicidal symptoms was determined by how participants scored this item. If a participant scored 0 (zero), it meant no symptom, whereas any score equal to or above 1 (1 to 3) signified the presence of suicidal ideation. Prevalence of suicidal ideation amongst elite soccer athletes was 46.73% (100 out of 214), and amongst controls was 22.73% (25 out of 110). The difference was statistically significant, χ^2^ = 17.663; *p* < 0.001; Cramér’s V = 0.223, indicating a small effect size, significantly lower prevalence of suicidal symptoms amongst controls when compared to elite soccer players.

**Figure 2:**
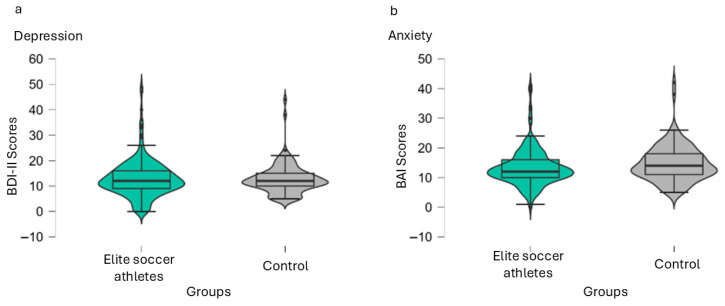
Violin elements graph separated by depression (**a**) and anxiety (**b**), showing sample distribution by group: general population age- and education-matched controls and elite soccer players. Note: BDI-II stands for Beck Depression Inventory 2nd edition. BAI stands for Beck Anxiety Inventory. Controls showed higher small effect-sized levels of anxiety in comparison to elite soccer athletes (*p* = 0.028; *r* = −0.166).

When considering levels of symptoms, instead of prevalence, depression yielded a non-significant difference between groups, U = 11,746.50; *p* = 0.977; *r* = −0.002. However, anxiety amongst controls showed higher levels when compared to elite soccer players with a small effect-size U = 9816.50; *p* = 0.028; *r* = −0.166 as revealed by the rank biserial correlation. Figure 2 depicts a violin-format distribution graph with median and 1.5 interquartiles of the medians for both depression and anxiety levels.

**Figure 3:**
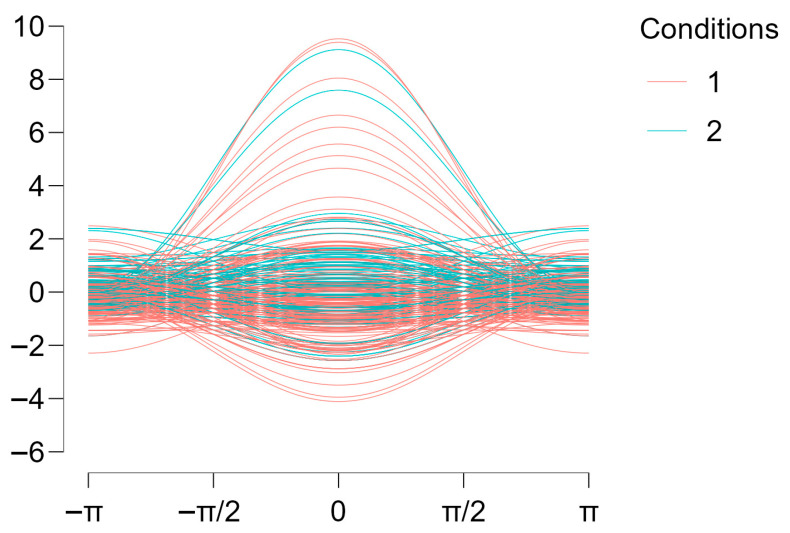
Andrews curves representing anxiety and depressive symptom patterns by group. Note: Andrews curves illustrating the joint distribution of Beck Anxiety Inventory (BAI) and Beck Depression Inventory-II (BDI-II) scores for elite soccer players and age- and education-matched controls. Each curve represents an individual participant, with the overall shape reflecting the combined symptom configuration. Curves are colour-coded by group (1 (red) = elite soccer players; 2 (blue) = controls). The horizontal axis represents the Andrews function index, and the vertical axis represents the corresponding transformed value derived from BAI and BDI-II scores. Overlapping trajectories indicate substantial similarity between groups, while systematic pattern variation reflects non-linear differences captured by the neural network classification.

The neural network model included two hidden layers with two nodes each and was trained on 260 participants, with performance evaluated on an independent test sample of 64 participants. Because group sizes differed (214 vs. 110), accuracy was interpreted alongside class-specific performance, as overall accuracy can be influenced by the majority class. The model achieved a test classification accuracy of 68.8%, indicating above-chance differentiation between elite soccer players and controls based on combined anxiety and depressive symptoms. However, the confusion matrix indicated that the accuracy reflected athletes’ sensitivity, while it showed no sensitivity for identifying controls, reflecting substantial overlap between groups. This confirms prior results that group overlap is substantial, and categorisation of this neural network could only be accounted for by sampling imbalance, not actual differences in mental health outcomes. Visual inspection of the Andrews curves suggested non-linear pattern differences despite overlapping symptom distributions (Figure 3). Overall, these findings indicate that anxiety and depressive symptoms jointly contain meaningful, though limited, information for group classification.

## 4. Discussion

This article aimed to investigate depression, anxiety and suicidal ideation prevalence and levels amongst elite soccer athletes in comparison to general population controls with similar demographics. Our study is unique because it identifies mental health outcomes amongst male elite soccer athletes with controls, differently from the previous study from Junge and Prinz [23], who studied female soccer athletes.

As initially hypothesised, there were no significant differences in depressive symptoms or prevalence of depression between elite soccer players and controls. This is consistent with previous studies that reported no statistical differences between controls and elite athletes [7,8,9,23]. However, it is still important to highlight that the nature of depressive symptoms amongst athletes might differ from that of the general population. Küettel and Larsen [3] demonstrated that the competitive nature of elite sports constitutes a unique environment that creates specific needs that, even though they might not seem to increase mental health symptoms, require unique care and practice. Because the sample comprises Brazilian first-division soccer players, contextual factors such as high public visibility, intense performance pressure, and organisational demands may shape symptom expression and help-seeking. Accordingly, generalisation beyond Brazilian elite football should be made cautiously and tested in other cultural contexts.

The same phenomenon occurred with the prevalence of anxiety. There was no significant difference between professional soccer players and controls. It is consonant with previous studies that found similar results [2,6]. Nonetheless, the level of anxiety symptoms in our results was higher amongst controls than elite soccer athletes, which might corroborate the study from Terry and Parsons-Smith [8].

Athletes face unique challenges, from pressure for results to excessive media exposure [4]. Even though athletes’ mental health seems unique from a phenomenological perspective, quantitatively, in previous studies [8] and in this study’s results, levels of anxiety symptoms were lower in professional soccer players than in controls from the general population. It might signify that the cognitive and emotional resources of elite athletes tend to differ from those of other people. Higher demands require higher adaptability, which might increase athletes’ resistance to anxiety symptoms [3,4,8]. From our perspective, the level of pressure and the competitive nature of elite sports might numb anxiety feelings amongst elite athletes.

In congruence with our initial hypothesis, suicidal ideation was less prevalent amongst controls when compared with elite soccer players. Age- and education-matched controls showed at least small suicidal ideation in 22.73% of participants, whereas elite soccer players were 46.73%. Previous prevalence studies suggested that athletes presented 7.6% to 18% of suicidal thoughts prevalence [10,11,12], whereas the general population had 3.3% to 11.5% [16,17,18,19]. However, it is essential to remember that these studies were conducted independently, which means they did not use the same methods. The only study with elite athletes and matched controls on suicidal ideation showed no significant difference in symptoms [14]; and yet, no study so far has shown the prevalence of suicidal thoughts utilising the agreement of the BDI-II item 9 amongst elite athletes and age- and education-matched controls [32].

Results found in this study on suicidal ideation prevalence are contrary to other results amongst collegiate athletes [13]. Our results suggest that elite soccer players might have distinct characteristics from NCAA athletes regarding suicidal thoughts. Sense of purpose, constant goal setting and changing, and increased sense of collectivism of team sports, might develop psychological resources to fight suicidal thoughts; however, our results point out that elite soccer players might face greater challenges such as external pressure, constant need for results and lack of social support, increasing their risk to suicidal ideation [3,4,13].

Regarding the neural network analysis, the moderate classification accuracy observed should be interpreted in light of the substantial overlap in anxiety and depressive symptom distributions between elite athletes and controls consistently reported in the literature [1,7,9]. In this context, a classification rate of 68.8% indicates that anxiety and depression jointly contain systematic information relevant to group differentiation, despite the absence of clear symptom boundaries. Nonetheless, the confusion matrix analysis revealed that, most likely, classification rates were due to sampling imbalance (i.e., 214 × 110) instead of actual accuracy in classification, which reinforces previous results from null-hypothesis testing that symptom levels between groups are non-significant. Similar classification magnitudes (63%) have been reported in recent neural network studies [36], which suggests that neural network analyses tend to fall around moderate above-chance accuracy when depression symptom severity is taken into consideration. Using the same methodology in prior research, cognitive and personality variables altogether yielded a significantly meaningful separation between elite athletes and non-athlete controls [26]. Rather than indicating weak performance, this study’s level of accuracy reflects the ecological reality of mental health phenomena, which are characterised by multidimensionality, co-morbidity, and above-chance affiliation to group membership. Accordingly, depression and anxiety were selected as inputs because both were assessed using full validated scales, whereas suicidal ideation was derived from a single item and was retained for prevalence comparisons rather than as a model feature to avoid circularity and over-weighting of a single indicator. This means modelling a single item based on a Likert-type scale of four categories with the same weight as a whole 21-item scale would create an unintended imbalance in statistical outcomes that we should avoid.

From a theoretical perspective, the present findings reinforce the argument that elite athletes cannot be reliably distinguished from the general population based on symptom severity alone. As previously suggested, elite sport environments may shape the expression and configuration of mental health symptoms rather than their absolute levels [3,4]. The neural network analysis supports this interpretation by showing that anxiety and depression interact in non-linear ways that modestly, but consistently, differentiate elite athletes from controls. This pattern mirrors findings reported by Bonnetti et al. [26], who demonstrated that neural network models using psychological and cognitive variables achieved even better classification performance, yielding high accuracy, providing meaningful insights into group-specific psychological organisation.

The present findings should be interpreted within a dimensional framework of mental health, rather than through categorical diagnostic classification. Both anxiety and depressive symptoms showed substantial overlap between elite soccer players and matched controls, a pattern consistently reported in elite sport research [1,7,9]. In this context, the use of continuous symptom measures allows for a more accurate representation of psychological functioning than diagnostic thresholds derived from cut-off points, which are known to reduce statistical power and obscure meaningful variability within and between groups [37,38]. This interpretation is further supported by the neural network analysis, which demonstrated that combined anxiety and depressive symptoms contributed to group differentiation in a probabilistic manner rather than through discrete classification boundaries. Similar approaches in elite sport research have shown that neural network models are particularly informative when applied to dimensional psychological constructs, even when categorical separation is limited [26]. Accordingly, mental health differences between elite athletes and the general population appear to be better characterised by variations in symptom configuration and severity than by rigid diagnostic categories.

In summary, our results suggest potential protective features related to lower anxiety severity among elite soccer players; however, these features do not appear to extend to suicidal ideation, which was more prevalent among athletes [8,13]. We found no statistical difference between athletes and controls in depression symptoms and prevalence, and anxiety prevalence [2,7,9,23]. It means that, even though professional soccer players feel the same amount of anxiety as the general population, the severity of symptoms is lower. Our results highlight the uniqueness of elite soccer athletes who seem to have protective psychological features that might reduce overall mental illness symptoms because of their ability to adapt to distressing situations.

### Limitations and Future Directions

One of the most relevant limitations of this study was the sampling method. Because the last author was a recognised sport psychology practitioner and researcher, a convenience sample was recruited through clubs’ sport psychologists and physical conditioning coaches. In addition, the study focused on male participants only to match available elite club sampling and to reduce heterogeneity in this first controlled comparison; future studies should replicate findings across genders. This means that a significant number of participants were athletes with psychological support in their respective organisations. In truth, no control group can fully capture the cultural and contextual specificity of elite Brazilian football; however, researchers in methodology of human sciences consistently recognises that such ideal controls are unattainable in human research, as in the words of the classical work of Campbell and Stanley [39]: “The true experiment may be defined as one in which there is control over all sources of invalidity. Such experiments are rare outside the laboratory”. In this study, matching controls on age and education (after inclusion and exclusion criteria were implemented) addresses two of the most robust determinants of mental health outcomes, thereby reducing major confounding influences while maintaining ecological validity under the substantial practical and ethical constraints inherent to elite sport research.

A dedicated sport psychologist might be able to tailor interventions that address mental health issues, enhancing athletes’ coping strategies and resilience [40,41]. That alone constitutes a confound variable not detailed in the present study. Future studies might want to look specifically at mental health outcomes amongst athletes with and without the support of a sport psychologist or other mental health practitioner to ensure that the effect we found in this article, congruent to previous literature, is not linked to the presence or absence of a mental health professional.

Another limitation was the criteria utilised to determine depression and anxiety prevalence. By using the minimal level in comparison to higher levels of these mental symptoms [42,43], we opted for a less sensitive approach to determine prevalence, which might have inflated our results. Accordingly, research on patients with hereditary angioedema revealed that a significant part of the sample experienced high levels of anxiety and depression, with BDI scores indicating that a notable percentage fell into the moderate to severe categories [44], which means that utilising severe levels of symptoms in contrast with lower levels might be an option for future studies amongst athletes such as done by Ojio et al. [12] and Timpka et al. [11].

Yet another limitation of the present study concerns the performance of the neural network classification, which, although above chance, yielded moderate accuracy when compared to other recent applications in elite sport. While Bonnetti et al. [26] reported near-perfect classification accuracy using neural networks to differentiate elite athletes based on cognitive and personality variables, their findings relied on a broader and more heterogeneous set of psychological inputs specifically selected to maximise group separability. In contrast, the present analysis was intentionally constrained to anxiety and depressive symptoms, constructs that are consistently shown to overlap substantially between elite athletes and the general population [1,7,9]. Although the model achieved above-chance classification, it was not intended for individual screening or diagnostic inference. The neural network was used as a confirmatory and supplementary method of classification to test whether combined symptom configurations carried probabilistic information about group affiliation. In this sense, the confusion matrix revealed that the most likely results of this modelling method were due to sampling imbalance rather than group distinction. Thus, any outputs should be interpreted at the group level to avoid stigmatising inferences about athletes’ mental health.

As demonstrated in mental health research using neural networks, moderate classification accuracy is common when models are applied to complex, multidimensional psychological phenomena rather than categorical diagnostic outcomes [36]. Therefore, the observed accuracy should be interpreted as reflecting the intrinsic phenomenological overlap of mental health symptoms across populations rather than methodological weakness. Future studies may benefit from integrating additional psychological, contextual, or sport-specific variables to examine whether classification outcomes improve accuracy without compromising ecological validity [3,4].

Finally, we decided to use item 9 of BDI-II that corresponds to suicidal ideation symptoms. Researchers argue that it is a valid way of measuring the prevalence of suicidal thoughts, and it is a time-effective tool because it belongs to a broader scale that is already being used [32]. We justified its use based on the fact that soccer, and elite sports overall, happen in a time-demanding environment and adding a third scale might have reduced the willingness of participants to collaborate with the research [3,23]. Nonetheless, previous studies such as Ojio et al. [12] and Jovanović et al. [14] managed to collect whole scales to measure suicidal ideation. We understand that the use of a single item is less precise than that of a whole scale and that it might have influenced our results. Future studies need to assess the necessity of including long or time-consuming scales in their data collection and balance the benefits and disadvantages of their choices.

Given the observed prevalence of suicidal ideation amongst elite soccer players, these findings have serious public health implications for the protection of athletes’ mental health. Systematic monitoring of anxiety, depressive symptoms, and suicidal ideation should be integrated into routine health and well-being surveillance in elite sport. This way, earlier identification of psychological risk might lead to a reduction in reliance on crisis-driven and urgent responses. From a public health perspective, monitoring should be embedded in clubs and sports organisations within clearly defined referral pathways, preferably with clinical psychologists and psychiatrists within the clubs, to ensure timely access to qualified mental health professionals and continuity of care beyond the sporting environment, particularly when higher risks are identified.

## 5. Conclusions

The present study addresses a relevant gap in elite soccer player mental health research. We compared anxiety, depression, and suicidal ideation among elite male soccer players and age- and education-matched controls from the general population. This research utilised standardised self-report measures, non-parametric group comparisons, and a supplementary neural network classification analysis. The results pointed out similar prevalence of anxiety and depression between groups, lower anxiety severity in athletes, and significantly higher suicidal ideation amongst elite soccer players. The neural network results further suggested that anxiety and depressive symptoms do not clearly discriminate athletes from controls, reinforcing the interpretation that these outcomes are better understood individually and contextually rather than as categorical markers of elite sports. For practitioners, either sport psychologists or psychiatrists, these findings highlight the need for routine mental health monitoring in elite soccer, with particular attention to suicide risk. Further implementation of structured referral pathways, or ongoing sports and clinical psychological support within high-performance soccer environments, is critical.

## Figures and Tables

**Table 1:** Descriptive statistics and Shapiro–Wilk normality test for age, education (in school years), depression and anxiety levels.

Variable	Groups	Sample (*N*)	Mean	95% CI		Std. Deviation	Shapiro–Wilk	
				Upper	Lower		Statistics	*p*-Value
Age	Athletes	214	25.29	25.82	24.77	3.90	0.97	<0.001
	Controls	110	25.25	26.07	24.42	4.37	0.93	<0.001
Education	Athletes	214	7.95	8.20	7.70	1.88	0.93	<0.001
	Controls	110	8.01	8.35	7.67	1.81	0.88	<0.001
Depression	Athletes	214	12.88	13.87	11.90	7.29	0.90	<0.001
	Controls	110	12.76	13.87	11.65	5.87	0.84	<0.001
Anxiety	Athletes	214	13.32	14.16	12.49	6.19	0.89	<0.001
	Controls	110	14.75	15.90	13.59	6.10	0.92	<0.001

**Table 2:** Severity of depressive and anxiety symptoms by group.

Mental Health Outcome	Group	Minimal *N* (%)	Mild *N* (%)	Moderate *N* (%)	Severe *N* (%)	Total *N*
Depressive symptom severity (BDI-II)						
	Elite soccer players	128 (59.81%)	61 (28.50%)	17 (7.94%)	8 (3.74%)	214
	Controls	69 (62.73%)	30 (27.27%)	9 (8.18%)	2 (1.82%)	110
Anxiety symptom severity (BAI)						
	Elite soccer players	27 (12.62%)	132 (61.68%)	47 (21.96%)	8 (3.74%)	214
	Controls	11 (10.00%)	55 (50.00%)	39 (35.45%)	5 (4.55%)	110

Note: Severity categories were based on established cut-off points for Brazilian samples: BDI-II (Minimal: 0–13; Mild: 14–19; Moderate: 20–28; Severe: ≥29) and BAI (Minimal: 0–7; Mild: 8–15; Moderate: 16–25; Severe: ≥26).

## Data Availability

Anonymized data is available at the Open Science Framework repository [35]. Data analysis was conducted in JASP 0.18.3, and syntaxes are available in the same repository under a different file (http://doi.org/10.17605/OSF.IO/TMVH9).
